# A Lightweight and Transparent Composite-Perforated Janus Acoustic Metamaterial: Multi-Band Sound Absorption Realized via Asymmetric Integrated Design

**DOI:** 10.3390/ma19153209

**Published:** 2026-07-27

**Authors:** Xinxin Liu, Guangjun Zhang, Jingbo Zhao, Runqing Zhang, Peizhou Hu

**Affiliations:** 1 Shaanxi Key Laboratory of Artificially Structured Functional Materials and Devices, Air Force Engineering University, Xi’an 710051, China; l2xlxx@163.com (X.L.); zhanggj3@126.com (G.Z.); 13227566559@163.com (R.Z.); hu496804307@163.com (P.H.); 2 Foundation Department, Air Force Engineering University, Xi’an 710051, China

**Keywords:** Janus acoustic metamaterial, labyrinthine helmholtz resonance, micro-perforated plate, low-frequency noise control, transparent SLA resin

## Abstract

**Highlights:**

- A transparent Janus acoustic metamaterial is designed with dual absorption mechanisms in one unit cell.
- Forward incidence gives near-unity absorption at 322 Hz (*α* > 0.8: 308–337 Hz).
- Reverse micro-perforated incidence shifts peak to 658 Hz with broader band (*α* > 0.8: 611–710 Hz).
- Front perforations tune Helmholtz frequency; rear micro-perforations enable independent band control.
- Theory, simulation, and experiments show excellent agreement, validating the numerical models.

**Abstract:**

To address the bottlenecks of conventional resonant sound-absorbing structures—namely, fixed frequency bands, single functionality, and inapplicability to light-limited spaces—this paper designs and fabricates a composite-perforated Janus acoustic metamaterial using transparent SLA resin. By integrating acoustic impedance theory, parametric finite-element simulations, and impedance-tube experiments, we systematically investigate the sound absorption mechanisms under forward and reverse incidences, as well as the regulatory effects of aperture diameters and spacing on both sides. Results demonstrate that under forward conventional perforation incidence, the structure achieves near-unity low-frequency narrowband absorption at 322 Hz, with coefficients exceeding 0.8 across 308–337 Hz. Under reverse micro-perforated incidence, the absorption peak shifts to 658 Hz with a substantially broader effective band, maintaining coefficients above 0.8 from 611 to 710 Hz. The close agreement among simulations, theory, and experiments validates the reliability of our numerical models. Parametric studies reveal that the front-side perforations primarily tune the Helmholtz resonance frequency with marginal impact on peak magnitude, whereas the rear-side micro-perforations exhibit high sensitivity to aperture size, enabling independent and flexible tuning of absorption bands via bilateral geometric decoupling. By integrating two distinct absorption mechanisms within a single unit cell, this Janus architecture overcomes the limitations of narrowband low-frequency performance and functional singularity. Coupled with its optical transparency, it offers a novel noise-control solution for light-transmitting equipment and industrial pipelines, while providing a valuable reference for the design of multifunctional asymmetric acoustic metamaterials.

## 1. Introduction

Low-frequency noise (LFN), a common acoustic pollutant widely existing in industrial pipelines, precision power equipment, and indoor public buildings, possesses inherent physical characteristics including long wavelength, slow attenuation, and strong penetrability. Numerous studies have proven that long-term exposure to LFN will cause chronic damage to the human auditory, nervous, and cardiovascular systems, whose harm is comparable to that of high-frequency noise at the same sound pressure level [1,2,3]. Traditional porous sound-absorbing materials such as foams and fibers exhibit excellent medium- and high-frequency sound dissipation performance [4]. Nevertheless, their sound absorption coefficient decreases sharply for acoustic waves below 1000 Hz, and extremely thick material thickness is required to achieve effective noise reduction, which is severely restricted by limited installation space in practical engineering [5].

Acoustic metamaterials based on Helmholtz resonance have become mainstream candidates for LFN control, owing to their unique advantage of low-frequency sound absorption with sub-wavelength thickness [6,7]. A classical Helmholtz resonator consists of a neck cavity and a resonant cavity. When the incident sound frequency matches the resonant frequency, fierce friction between high-speed airflow and cavity wall converts acoustic energy into thermal energy for energy dissipation [8,9]. However, conventional Helmholtz resonators suffer from narrow absorption bandwidth and low tuning freedom, limiting their application in complex noise environments [10,11]. To solve such drawbacks, various modified resonant structures have been developed. Duan et al. [12] designed a multi-layer Helmholtz resonant metamaterial to generate multiple low-frequency absorption peaks via series-connected cavities. Zhang et al. [13] proposed a resonator with fully rough embedded neck to improve low-frequency acoustic performance by optimizing neck morphology. Xu et al. [14] investigated the acoustic characteristics of dual Helmholtz resonators, which effectively broadened the effective sound-absorbing bandwidth.

In recent years, Janus asymmetric acoustic metamaterials have realized bidirectional differentiated acoustic responses via asymmetric topological configuration, providing a novel route for flexible noise regulation under complex working conditions [15,16,17,18,19]. Chuan et al. [20] explored the asymmetric transmission and focusing performance of acoustic metamaterials; Guo et al. [21] achieved switchable bidirectional sound absorption via metasurface modulation; Gu et al. [22] developed a double-layer piezoelectric asymmetric metamaterial for low-frequency broadband noise attenuation. Nevertheless, most reported Janus structures adopt simple single-cavity design with deficiencies including insufficient low-frequency absorption capacity, limited parameter tunability, and poor light transmittance, failing to integrate low-frequency efficient absorption, broadband noise reduction, and optical transparency simultaneously [23]. In terms of manufacturing materials, most existing acoustic metamaterials are fabricated by metals or opaque polymers, which cannot be applied to daylighting curtain walls, observation pipelines, and transparent equipment cabins that require both light transmission and noise suppression [24,25,26,27,28]. Conventionally, LFN attenuation heavily relies on high-density materials following the mass law. Representative strategies include double-panel mass-spring-mass partitions, constrained layer damping structures, high-mass-loaded polymer sheets, and inertial mass blocks for structure-borne vibration isolation. Nevertheless, these classic approaches inevitably sacrifice optical transparency and usually introduce excessive structural weight, restricting their deployment in light-transmitting facilities. Transparent acoustic metamaterials are emerging rapidly, and existing studies indicate that multifunctional integration and optical transparency are core research orientations for future engineering applications [29].

**Figure 1:**
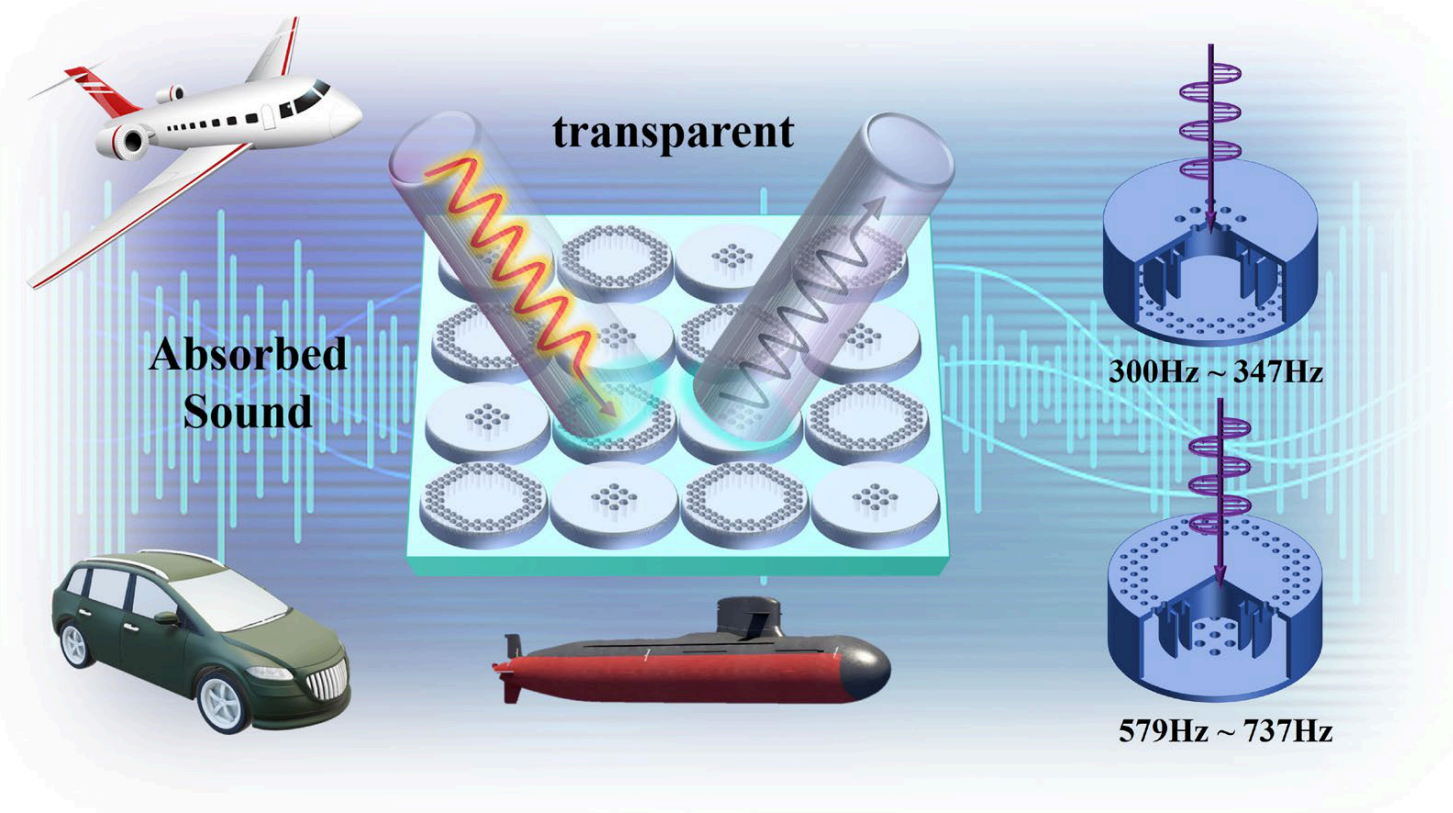
Schematic diagram of this work: transparent composite-perforated Janus acoustic metastructure, which achieves bidirectional differentiated acoustic responses via front-back asymmetric configuration.

Despite the great progress of acoustic metamaterials, three key issues still need to be addressed: (1) integrating multiple sound-absorbing mechanisms in a single unit to overcome the defects of single function and poor low-frequency performance of traditional absorbers; (2) balancing optical transparency and low-frequency acoustic performance to realize noise control in daylighting confined spaces; and (3) realizing independent modulation of bidirectional absorption bands to adapt to variable complex noise conditions. Targeting the above problems, this work designs and fabricates a composite-perforated Janus annular acoustic metamaterial via transparent stereolithography (SLA) resin 3D printing technology. Janus acoustic metamaterials are defined as artificial metastructures with direction-asymmetric acoustic properties. As illustrated in Figure 1, the proposed integrally molded structure is suitable for LFN suppression in daylighting-required confined spaces, including aircraft, submarines, and automobiles. It consists of a top nine-hole conventional perforated panel, central cylindrical coupling cavities, multi-stage annular labyrinth Helmholtz channels, and a bottom annular micro-perforated panel, which delivers a unique bidirectional asymmetric acoustic response. In practical applications, the staggered arrangement of structural units on the front and rear surfaces of the sample enables the structure to realize effective broadband sound absorption. Combining acoustic impedance theory, parametric finite-element simulation, and impedance-tube experimental tests, the bidirectional sound absorption mechanism and parameter modulation rules of perforation size and spacing are investigated systematically. This study provides a feasible scheme for low-frequency noise reduction in daylighting confined spaces, and offers theoretical support and engineering reference for the design optimization of multifunctional asymmetric acoustic metamaterials [30].

## 2. Structure Design

**Figure 2:**
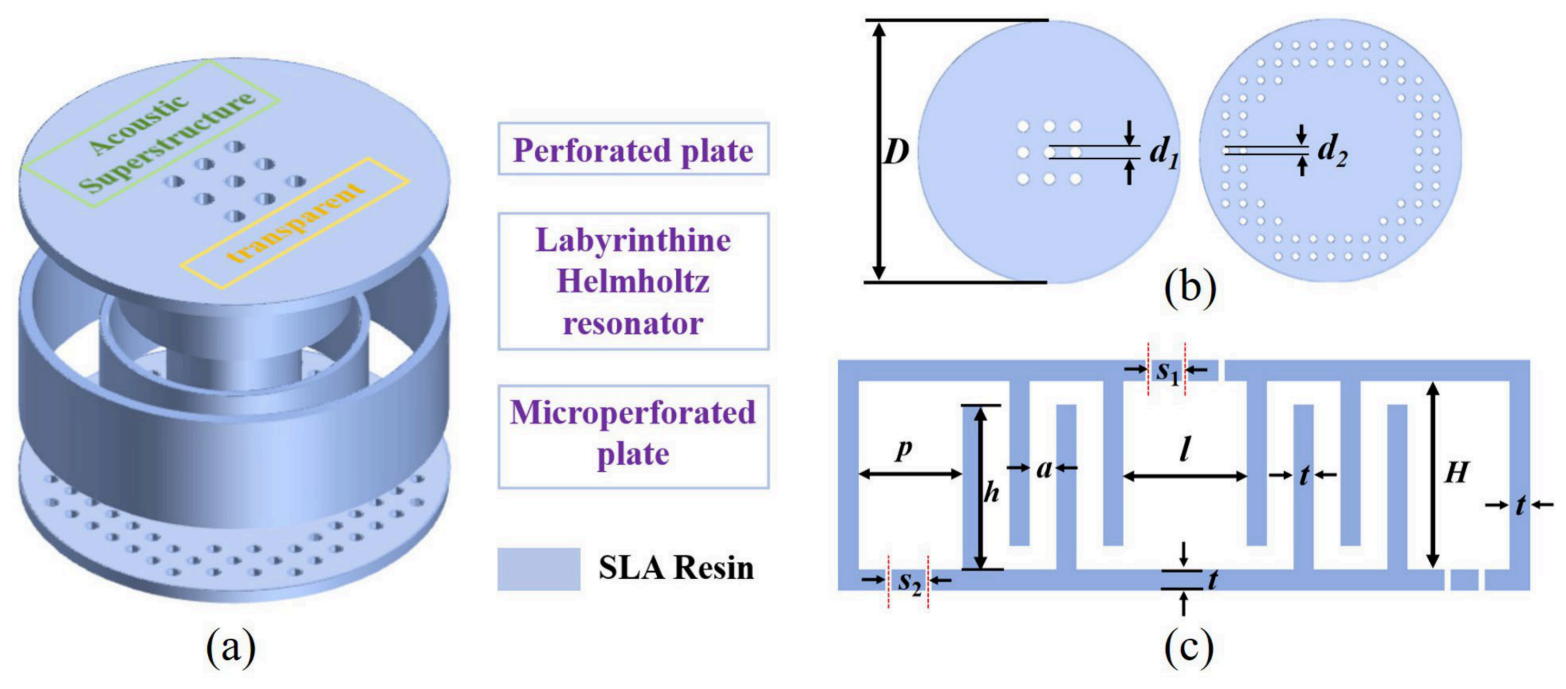
Design architecture of the acoustic unit. (**a**) Overall design principle; (**b**) top view of the upper and lower surfaces of the acoustic unit; and (**c**) cross-sectional view of the cavity architecture.

The schematic diagram of the proposed composite-perforated Janus acoustic metamaterial is displayed in Figure 2. The metamaterial adopts an integral cylindrical configuration with an outer diameter D of 99 mm, cylinder wall thickness t of 3 mm, and total cylinder height of 21 mm. An open-ended labyrinth channel is arranged inside the cylinder, which is assembled by four orderly arranged annular plates n=4. The geometric parameters of annular plates are defined as: plate height h = 12 mm, gap spacing a = 3 mm, plate thickness t = 3 mm. One end of the labyrinth channel communicates with the central cylindrical cavity, while the other end is connected to the outer annular cavity. The central cylindrical cavity has an inner diameter l=27 mm and height H = 15 mm. Nine uniform short pipes are arranged at the upper opening of the central cavity to form a conventional perforated panel, with perforation diameter d1 = 1.6 mm and hole spacing $S_{1}$ = 7.1 mm. The outer annular cavity has a cavity width p = 12 mm, and its bottom opening is equipped with an annular micro-perforated panel. The micro-perforated panel owns a perforation diameter d2 = 1 mm and hole spacing $S_{2}$ = 6.2 mm.

**Figure 3:**
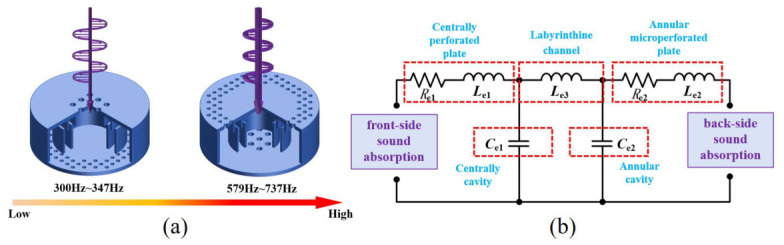
Analysis of the acoustic unit. (**a**) Schematic diagram of overall acoustic effect and (**b**) equivalent electro-acoustic circuit of the acoustic unit.

The proposed composite-perforated Janus metastructure is fabricated from transparent SLA resin to achieve optical transparency. The transparent SLA resin is a commercial UV-curable acrylate-based photopolymer, mainly consisting of polyurethane acrylate oligomer, reactive acrylic diluents, and phosphine oxide photoinitiator, and detailed material parameters are listed in Table 1. Meanwhile, the overall ultrathin structural design endows it with a lightweight and aesthetic appearance for practical application. The effective sound-absorbing thickness of the structure is merely about 2 cm, and favorable noise absorption performance can be realized via partial lightweight layout. Benefiting from the transparent feature, the operating state of engineering equipment can be directly observed, which facilitates the inspection of internal dust accumulation and equipment faults, solving the visual inspection limitation of traditional metal mufflers. Furthermore, by combining the labyrinth channel design, the perforated panel layout, and the asymmetric Janus configuration, the metastructure achieves differentiated sound-absorbing performances on two opposite sides—as illustrated in Figure 3a—which greatly improves its overall acoustic performance. The embedded labyrinth channel makes full use of internal cavity space to extend the airflow propagation path, which acts on low-frequency sound waves, reduces the resonant absorption frequency, and optimizes the low-frequency sound absorption capacity.

## 3. Theoretical Analysis

Based on acoustic fundamentals and the electro-acoustic analogy method, relative motion occurs between fluid medium and pipe wall inside short branch pipes under acoustic excitation, producing viscous resistance. The viscous effect consumes acoustic energy, which is defined as acoustic resistance element R analogous to circuit resistance $R_{e}$. The fluid medium inside branch pipes and labyrinth channels is regarded as acoustic mass element M analogous to circuit inductance $L_{e}$. The air medium in cavities has elastic properties, and the resonant cavity is simplified as acoustic capacitance element C analogous to circuit capacitance $C_{e}$. The equivalent electro-acoustic circuit of the Janus metastructure is obtained [31], as shown in Figure 3b.

For the central perforated panel, its acoustic impedance can be expressed as follows [31]:

(1) $$Z_{CPP}=R_{CPP}+j\omega M_{CPP}+Z_{CC}$$

where $Z_{CC}$ denotes the acoustic impedance of the central cavity. The real part of the acoustic impedance of the central perforated panel $R_{CPP}$ originates from the viscous effect between air and inner pipe wall, while its imaginary part $\omega M_{CPP}$ reflects the inertial motion of air inside perforation holes. The above parameters can be defined by Equations (2)–(5) [32]:

(2) $$R_{\mathrm{CPP}}=\frac{\sqrt{2\rho_{0}\mu \omega }}{\xi_{s}}\left(\frac{2t}{d_{1}}+2\right)$$

(3) $$M_{CPP}=\frac{\rho_{0}\left(t+0.85d_{1}\right)}{\xi_{s}}$$

(4) $$Z_{CC}=-j\rho_{0}c_{0}\cot\left(\frac{\omega H}{c_{0}}\right)$$

(5) $$\xi_{s}=\frac{9\pi {d_{1}}^{2}/4}{\pi l^{2}/4}$$

where $M_{CPP}$ represents the acoustic mass of the central perforated panel, μ is the viscosity coefficient of the air medium, ω=2πf refers to the angular frequency, $\xi_{s}$ is the ratio of perforation area to the equivalent area of the central perforated sound-absorbing unit, $\rho_{0}$ denotes air density, and $c_{0}$ is the speed of sound.

To calculate the acoustic impedance of the two-end open labyrinth channel, relevant parameters are defined as follows [33]:

(6) $$\left\{\begin{array}{c} k_{n}=-j\omega \frac{\rho_{0}}{\mu }\\ k_{r}=-j\omega \frac{\rho_{0}C_{p}}{K} \end{array}\right.$$

where *K* is the fluid thermal conductivity, $C_{p}$ denotes the constant-pressure specific heat capacity, $k_{n}$ is the viscous wave number, and $k_{r}$ represents the thermal wave number. Considering the viscous field $\psi_{n}$ and thermal field $\psi_{r}$, the equivalent parameters of the labyrinth channel after thermal-viscous coupling can be further derived as follows [33]:

(7) $$\left\{\begin{array}{c} k_{f}={k_{0}}^{2}\left[\frac{\gamma -\left(\gamma -1\right)\psi_{r}}{\psi_{n}}\right]\\ \rho_{c}=\frac{\rho_{0}}{\psi_{n}}\\ \begin{array}{l} c_{c}=\frac{\omega }{k_{f}}\\ k_{c}=\sqrt{k_{f}} \end{array} \end{array}\right.$$

where $k_{0}=\omega /c_{0}$ is the acoustic wave number in air, $k_{f}$ is the equivalent complex wave number, $k_{c}$ is the intermediate equivalent wave number, $\rho_{c}$ is the complex air density, $c_{c}$ is the intermediate equivalent sound velocity, and γ is the heat capacity ratio of air. The acoustic impedance of the two-end open labyrinth channel can be expressed as follows [34]:

(8) $$Z_{LC}=j\frac{\rho_{c}c_{c}}{{\alpha_{s}}^{2}}\tan\left(k_{c}D_{y}\right)$$

where $D_{y}$ represents the effective propagation length of sound waves inside the labyrinth channel, $\alpha_{s}$ is the cross-section scaling coefficient, $\alpha_{s}=S_{1}/S_{0}$, $S_{1}$ denotes the cross-sectional area of inner labyrinth slit, and $S_{0}$ is the inlet cross-sectional area for sound incident into the labyrinth channel.

For the annular micro-perforated panel, its acoustic impedance can be expressed as follows [31]:

(9) $$Z_{AMPP}=R_{AMPP}+j\omega M_{AMPP}+Z_{AC}$$

where $Z_{AC}$ denotes the acoustic impedance of the annular cavity. The real part of the acoustic impedance of the annular micro-perforated panel $R_{AMPP}$ derives from the viscous effect between air and inner hole wall, while its imaginary part $\omega M_{AMPP}$ reflects the inertial motion of air inside micro-perforations. These parameters can be calculated by Equations (10)–(13) [31]:

(10) $$Z_{AC}=-j\rho_{0}c_{0}\cot\left(\frac{\omega H}{c_{0}}\right)$$

(11) $$R_{AMPP}=\frac{32\mu t}{\sigma {d_{2}}^{2}}\left[\sqrt{1+\frac{k^{2}}{32}}+\frac{\sqrt{2}}{8}\frac{kd_{2}}{t}\right]$$

(12) $$M_{AMPP}=\frac{\rho_{0}t}{\sigma }\left[1+{\left(9+\frac{k^{2}}{2}\right)}^{-\frac{1}{2}}+0.85\frac{d_{2}}{t}\right]$$

(13) $$k=d_{2}\sqrt{\omega \rho_{0}/4\mu }$$

where $M_{AMPP}$ represents the acoustic mass of the structure, and $\sigma =\pi {d_{2}}^{2}/4{S_{2}}^{2}$ is the porosity of the micro-perforated panel.

**Figure 4:**
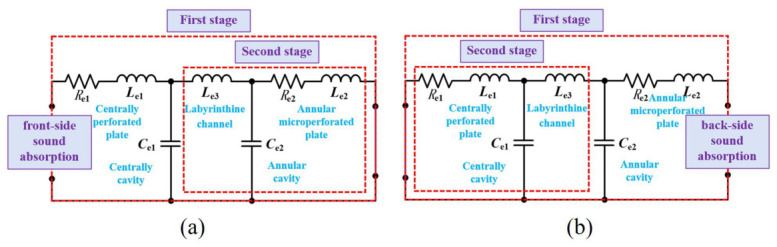
Hierarchical diagram of equivalent circuits. (**a**) Hierarchical equivalent circuit for front sound absorption and (**b**) hierarchical equivalent circuit for back sound absorption.

According to Figure 4a, the proposed metastructure can be regarded as a two-stage series-parallel structure when sound waves are incident from the front side. The second-stage structure is a parallel configuration composed of the two-end open labyrinth channel series-connected with the annular micro-perforated panel and annular cavity. The first-stage structure is a parallel configuration consisting of the central perforated panel series-connected with the central cavity and the second-stage structure. The above two-stage structures constitute the integral metastructure. Based on the series and parallel principles of acoustic impedance [35], the acoustic impedance expressions are given as follows [32]:

(14) $$Z_{FS\_2}=Z_{LC}+Z_{AC}-\frac{{Z_{AC}}^{2}}{R_{AMPP}+j\omega M_{AMPP}+Z_{AC}}$$

(15) $$Z_{FS\_1}=R_{CPP}+j\omega M_{CPP}+\frac{Z_{FS\_2}Z_{CC}}{Z_{FS\_2}+Z_{CC}}$$

Combining the relationship between sound absorption coefficient $\alpha_{FS}$ and acoustic impedance $Z_{FS\_1}$, the front sound absorption coefficient of the metastructure can be obtained as follows [36]:

(16) αFS=1−ZFS_1−Z0ZFS_1+Z02=4Re(ZFS_1/Z0)1+Re(ZFS_1/Z0)2+Im(ZFS_1/Z0)2

where the acoustic impedance $Z_{0}=\rho_{0}c_{0}$ refers to the characteristic acoustic impedance of the air medium.

According to Figure 4b, the metastructure can also be regarded as a two-stage series-parallel structure when sound waves are incident from the back side. The second-stage structure is a parallel configuration composed of the two-end open labyrinth channel series-connected with the central perforated panel and central cavity. The first-stage structure is a parallel configuration consisting of the annular micro-perforated panel series-connected with the annular cavity and the second-stage structure. The above two-stage structures constitute the integral metastructure. Based on the series and parallel principles of acoustic impedance, the acoustic impedance expressions are given as follows:

(17) $$Z_{BS\_2}=Z_{LC}+Z_{CC}-\frac{{Z_{CC}}^{2}}{R_{CPP}+j\omega M_{CPP}+Z_{CC}}$$

(18) $$Z_{BS\_1}=R_{AMPP}+j\omega M_{AMPP}+\frac{Z_{BS\_2}Z_{AC}}{Z_{BS\_2}+Z_{AC}}$$

Similarly, combined with the correlation between sound absorption coefficient $\alpha_{BS}$ and acoustic impedance $Z_{BS\_1}$, the back sound absorption coefficient of the metastructure can be acquired as follows [34]:

(19) αBF=1−ZBS_1−Z0ZBS_1+Z02=4Re(ZBS_1/Z0)1+Re(ZBS_1/Z0)2+Im(ZBS_1/Z0)2

## 4. Finite-Element Simulation Analysis

To investigate the sound absorption performance of the proposed metastructure, finite-element simulation is carried out via the Acoustic-Structure Interaction module in COMSOL Multiphysics 6.2 software. The composite-perforated Janus metastructure is made of transparent SLA resin in the numerical model, and detailed material parameters are listed in Table 1. Material parameters used in experiments and simulations were measured from SLA resin specimens. All test samples were manufactured with consistent printing orientation, layer thickness, exposure settings, temperature, and post-curing conditions. Although SLA-printed materials are strongly dependent on fabrication parameters and may display nonlinear characteristics under large strain, the structure is subjected to small-amplitude acoustic excitation in this research. Hence, constant linear material properties are utilized for the linear acoustic finite element analysis. During the simulation, fixed constraint boundary conditions are applied to the outer structural frame to eliminate the vibration interference of the shell frame. Meanwhile, the Thermoviscous Acoustics physics field is added to fully consider the thermal and viscous dissipation effects inside acoustic perforations and channels. Free tetrahedral elements are utilized for spatial discretization. The maximum element size is strictly limited below λ/6 at the upper-limit frequency to guarantee calculation precision. A systematic mesh refinement study is carried out with three sets of mesh densities. When further refining the mesh, the variation of calculated absorption coefficient is less than 1%, confirming the achievement of mesh convergence. A direct frequency domain solver is employed. Frequency sweep is performed from 50 Hz to 1000 Hz with a discrete frequency step of 2 Hz. The relative residual convergence criterion is set to $10^{-4}$.

**Figure 5:**
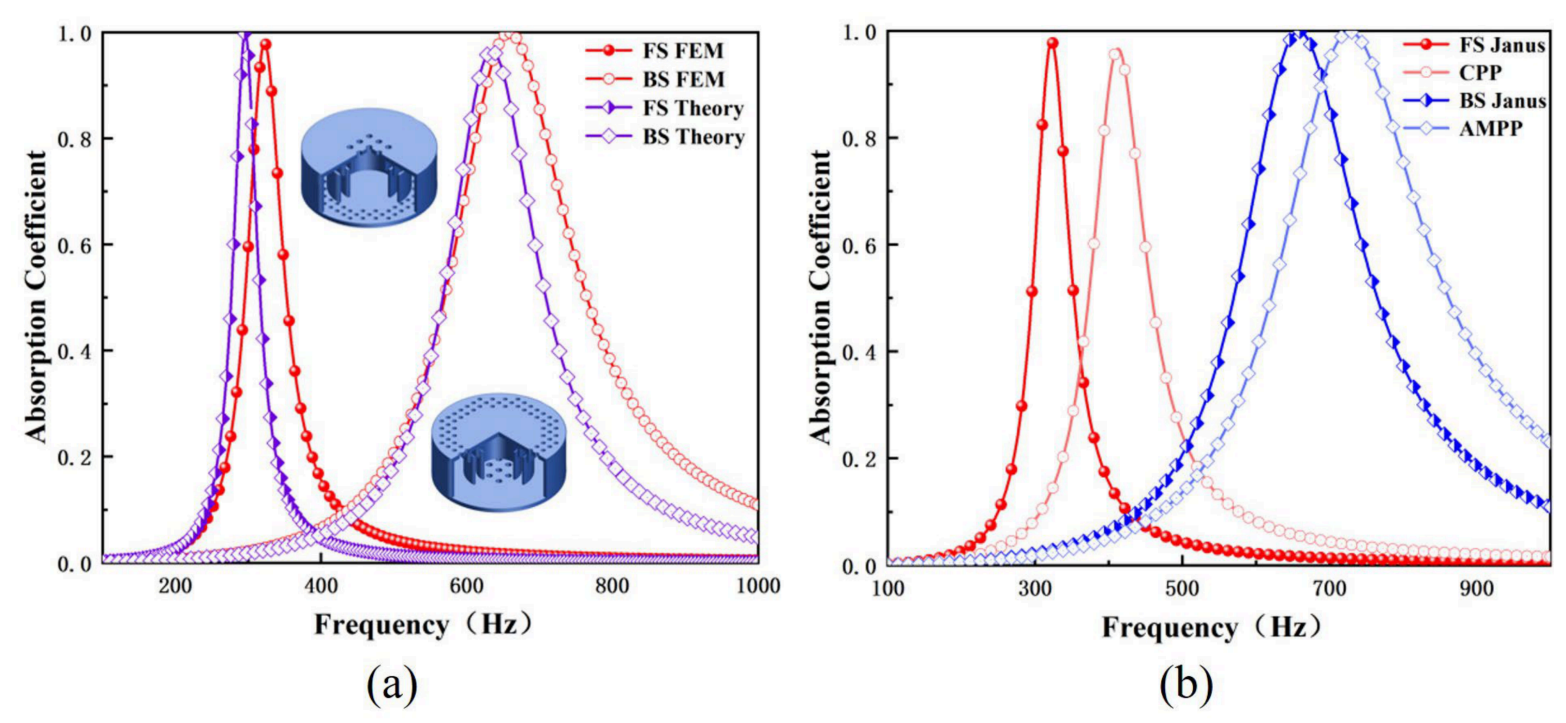
Curves of sound absorption coefficient. (**a**) Comparison between finite-element simulation and theoretical results and (**b**) performance comparison between the proposed Janus acoustic metastructure and traditional structures.

The simulated sound absorption coefficient curves are depicted in Figure 5a. The mutual verification between theoretical analytical solutions and finite-element simulation results confirms the reliability of the bidirectional sound absorption law of the designed Janus acoustic metastructure. For front sound incidence, efficient narrowband low-frequency sound absorption is achieved around 322 Hz, and the sound absorption coefficient exceeds 0.8 within the frequency range of 308–337 Hz. For back sound incidence, broadband sound absorption is obtained at 658 Hz, and the sound absorption coefficient is higher than 0.8 in the range of 611–710 Hz. The bidirectional differentiated sound absorption effect fully verifies the acoustic regulation characteristics of the asymmetric configuration. There is a certain discrepancy in peak frequencies between the theoretical predictions and finite-element-method (FEM) simulations. This is mainly attributed to the homogeneous equivalent medium approximation adopted in the theoretical model, which ignores local resonances, higher-order modes, acoustic scattering, and propagation path distortion inside cavities and bent channels. In contrast, FEM can resolve the detailed internal geometry of the structure and capture local wave effects. Such idealized assumptions in the equivalent model constitute the primary source of peak frequency shift.

Moreover, the acoustic performance of the proposed structure is compared with traditional central perforated panel (CPP) and annular micro-perforated panel (AMPP). Figure 5b reveals that traditional sound-absorbing structures merely realize unidirectional noise reduction in a single frequency band, with fixed functional modes and limited low-frequency absorption capacity. Multiple structures need to be combined for multi-working-condition noise control, which occupies large installation space and presents poor regulation flexibility. Benefiting from the asymmetric perforation layout and coupled resonance configuration of multi-stage annular labyrinth channels, the proposed Janus metastructure integrates two noise-reduction mechanisms, namely Helmholtz resonance low-frequency absorption and micro-perforation thermoviscous broadband absorption, in a single unit to realize bidirectional acoustic function switching. Compared with traditional structures, the proposed Janus metastructure extends the low-frequency absorption limit under the same overall dimension and possesses dual parameter regulation degrees of freedom. The sound absorption frequency bands on two sides can be independently tuned by optimizing front and rear perforation parameters, which meets the engineering demands of low-frequency noise reduction and broadband noise suppression. This design reduces the installation space and manufacturing cost of noise reduction devices and provides a novel technical idea for the design of multifunctional acoustic metamaterials in confined spaces.

**Figure 6:**
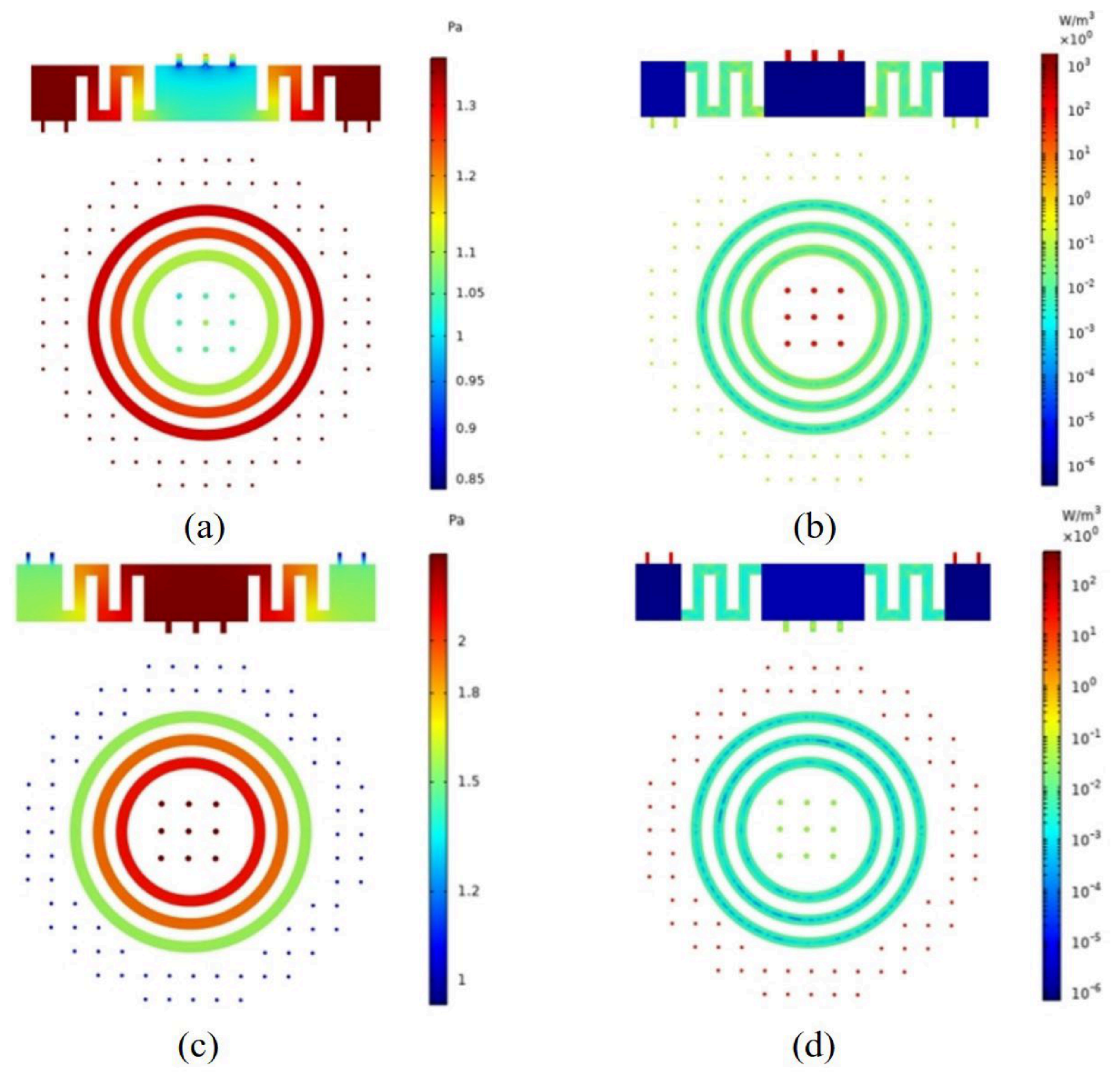
Simulation nephograms at sound absorption peak frequencies. (**a**) Sound pressure nephograms of axial cross-section and top-view annular cross-section under front incidence; (**b**) power dissipation density nephograms of axial cross-section and top-view annular cross-section under front incidence; (**c**) sound pressure nephograms of axial cross-section and top-view annular cross-section under back incidence; and (**d**) power dissipation density nephograms of axial cross-section and top-view annular cross-section under back incidence.

Numerical simulations are conducted when sound waves are incident from the upper and lower surfaces of the Janus metastructure, respectively. The axial cross-section and top-view annular cross-section nephograms of sound pressure and power dissipation density at absorption peak frequencies are obtained, as shown in Figure 6.

According to Figure 6a,b, when sound waves are incident frontally from the upper central perforated panel, nine circumferentially uniform perforations and the rear central cylindrical cavity form a Helmholtz resonance system. The air columns at perforation necks perform piston-type reciprocating vibration and generate intense thermoviscous dissipation, which serves as the primary acoustic energy consumption region at the corresponding resonant frequency. After being attenuated by the perforated panel, incident sound waves enter the central cylindrical cavity for uniform circumferential shunting and then flow into the annular labyrinth channels in an orderly manner. Sound pressure is highly concentrated inside the annular labyrinth, and secondary acoustic energy dissipation is generated relying on the air column resonance of labyrinth channels. The attenuated sound waves propagate to the bottom annular micro-perforated area, where transmitted sound pressure with a certain amplitude can be observed, but no resonant energy aggregation occurs, resulting in only weak auxiliary energy dissipation.

As depicted in Figure 6c,d, the acoustic field distribution changes significantly when sound waves are incident backward from the bottom annular micro-perforated panel. Sound pressure is mainly concentrated in the central cylindrical cavity and annular labyrinth channels. The neck area of the bottom annular micro-perforated panel replaces the top central perforations as the core energy dissipation unit, and the sound absorption process is dominated by thermoviscous loss of micro-perforations, while the energy dissipation intensity of outer labyrinth channels decreases obviously. In conclusion, the proposed Janus acoustic metastructure presents bidirectional asymmetric acoustic response characteristics. For frontal incidence from the central perforated panel, perforation-based Helmholtz resonance acts as the dominant dissipation mechanism, and the annular labyrinth provides auxiliary energy consumption, which adapts to low-frequency noise attenuation. For backward incidence from the annular micro-perforated panel, micro-perforation thermoviscous loss dominates acoustic energy consumption to achieve broadband sound absorption. Meanwhile, the integrated annular configuration enables uniform circumferential acoustic excitation without acoustic incident blind zones. The synergistic effect of multiple energy dissipation mechanisms effectively optimizes the overall sound absorption performance of the structure.

## 5. Numerical Study on Structural Parameter Design

**Figure 7:**
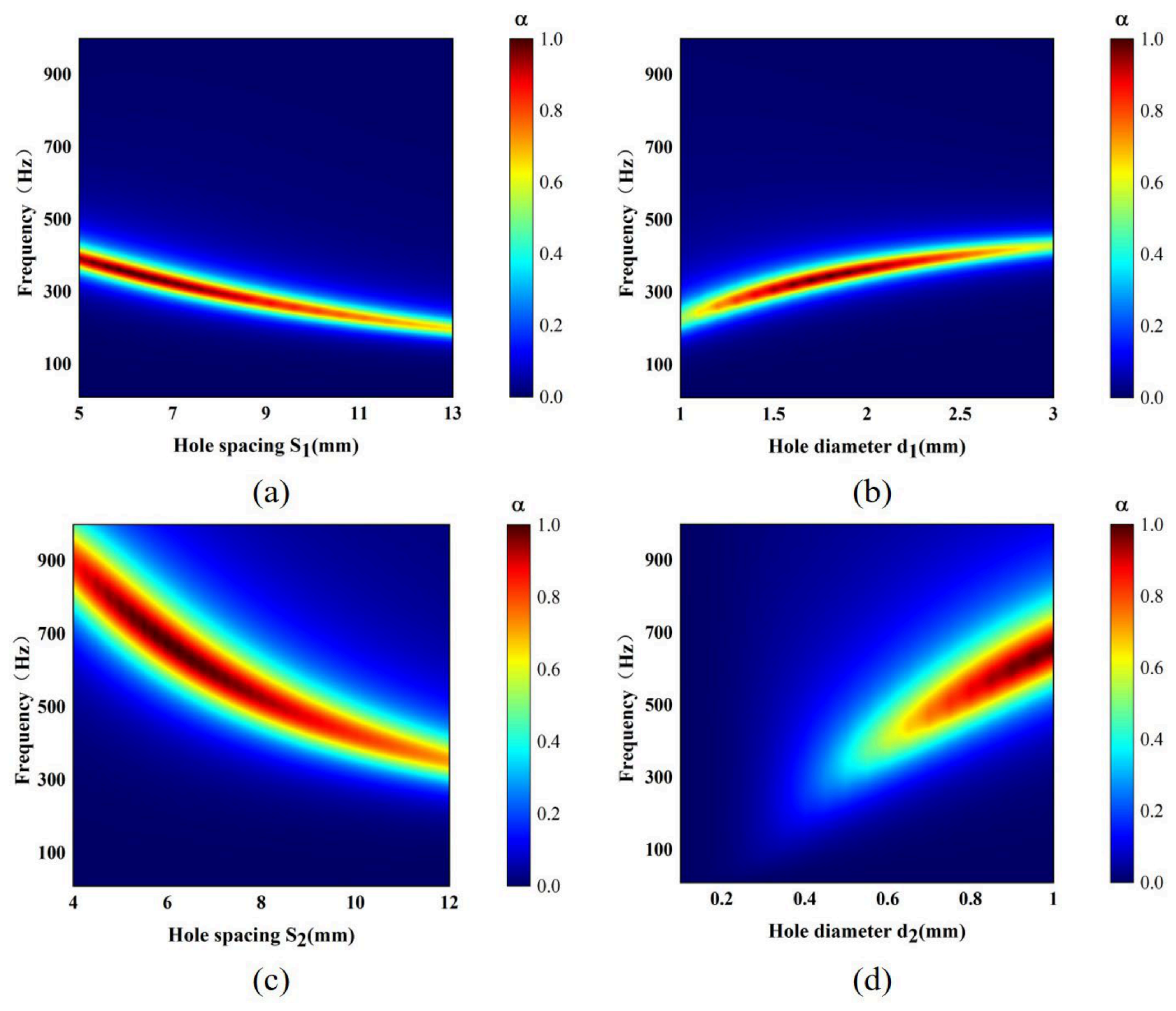
Structural parameter contour maps. (**a**) Frequency evolution law of sound absorption coefficient α affected by hole spacing $S_{1}$ of central perforated panel under front incidence; (**b**) frequency evolution law of sound absorption coefficient α affected by hole diameter $d_{1}$ of central perforated panel under front incidence; (**c**) frequency evolution law of sound absorption coefficient α affected by hole spacing $S_{2}$ of annular micro-perforated panel under back incidence; and (**d**) frequency evolution law of sound absorption coefficient α affected by hole diameter $d_{2}$ of annular micro-perforated panel under back incidence.

To further explore the parametric design law of the proposed metastructure, contour maps characterizing the correlation between structural parameters and sound absorption performance are plotted via the control-variable method, as shown in Figure 7. The four groups of contour maps illustrate the frequency-dependent evolution characteristics of sound absorption coefficient α affected by four key structural parameters, namely the hole spacing $S_{1}$ and hole diameter $d_{1}$ of the front central perforated panel, as well as the hole spacing $S_{2}$ and hole diameter $d_{2}$ of the back annular micro-perforated panel. In the contour nephograms, the red area corresponds to the region with high sound absorption coefficient, which can intuitively reflect the variation rules of absorption peak frequency and effective absorption bandwidth under different structural parameter configurations.

For the front sound incidence condition, when the hole spacing $S_{1}$ of the central perforated panel increases from 5 mm to 13 mm, the panel porosity decreases gradually. Correspondingly, the sound absorption peak frequency shifts toward the low-frequency region, the high-efficiency sound absorption bandwidth narrows slightly, and the peak sound absorption coefficient declines marginally. When the perforation hole diameter $d_{1}$ increases from 1 mm to 3 mm, the flow acoustic resistance at perforation necks decreases, the structural resonant frequency moves gradually to the mid-to-high frequency range, the absorption peak value increases continuously, and the effective sound absorption bandwidth is broadened. An excessively small perforation aperture will generate excessive acoustic damping, triggering interface reflection of most incident sound waves. In this case, sound waves can hardly couple into the internal resonant cavity, resulting in an overall low sound absorption coefficient in the low-frequency range.

For the back micro-perforation incidence condition, the hole spacing $S_{2}$ of the annular micro-perforated panel is also negatively correlated with the sound absorption peak frequency. Larger hole spacing corresponds to lower porosity, lower resonant peak frequency, and narrower effective absorption bandwidth. Compared with the front conventional perforated structure, the sound absorption performance of the back micro-perforated structure is more sensitive to the variation of hole spacing. The micro-perforation hole diameter $d_{2}$ presents an obvious nonlinear regulation effect on acoustic performance. When the aperture is smaller than 0.4 mm, excessive air thermoviscous dissipation occurs inside micro-pores, which hinders sound wave transmission into the cavity and weakens the overall sound absorption capacity. With the continuous increase of aperture size, the absorption peak frequency rises gradually, and the peak absorption coefficient improves remarkably. Wideband high-efficiency sound absorption can be achieved within the aperture range of 0.8–1.0 mm.

As mentioned above, the proposed composite-perforated Janus acoustic metastructure possesses great flexibility in acoustic performance modulation. Targeted design of sound absorption peaks and tunable absorption frequency bands can be realized by optimizing structural geometric parameters. In general, the front conventional perforation parameters optimize low-frequency sound absorption performance by regulating Helmholtz resonant frequency, and parameter variation exerts a mild influence on the sound absorption coefficient. The back micro-perforated structure realizes broadband sound absorption relying on air thermoviscous dissipation, and its acoustic performance is highly sensitive to aperture parameters. The differentiated parameter regulation rules of the two sides further explain the intrinsic bidirectional asymmetric acoustic response of the designed Janus metastructure.

## 6. Experimental Validation

Physical experiments are conducted to validate the effectiveness of the equivalent electro-acoustic model and the accuracy of numerical calculation. With the rapid maturity of 3D printing technology in recent years, high-precision fabrication of acoustic test specimens has become feasible. Firstly, the physical prototype of the proposed Janus metastructure is manufactured via 3D printing technology. Subsequently, the AWA6290T transfer-function measurement system is adopted to test the sound absorption coefficient of the prototype within the frequency range of 50–1000 Hz. The whole test platform consists of an impedance tube, two precision microphones, a power amplifier, and a signal analyzer. The measurement principle follows the two-microphone transfer-function method specified in ISO 10534-2 [37]. The experimental data are recorded and further compared with numerical simulation results.

**Figure 8:**
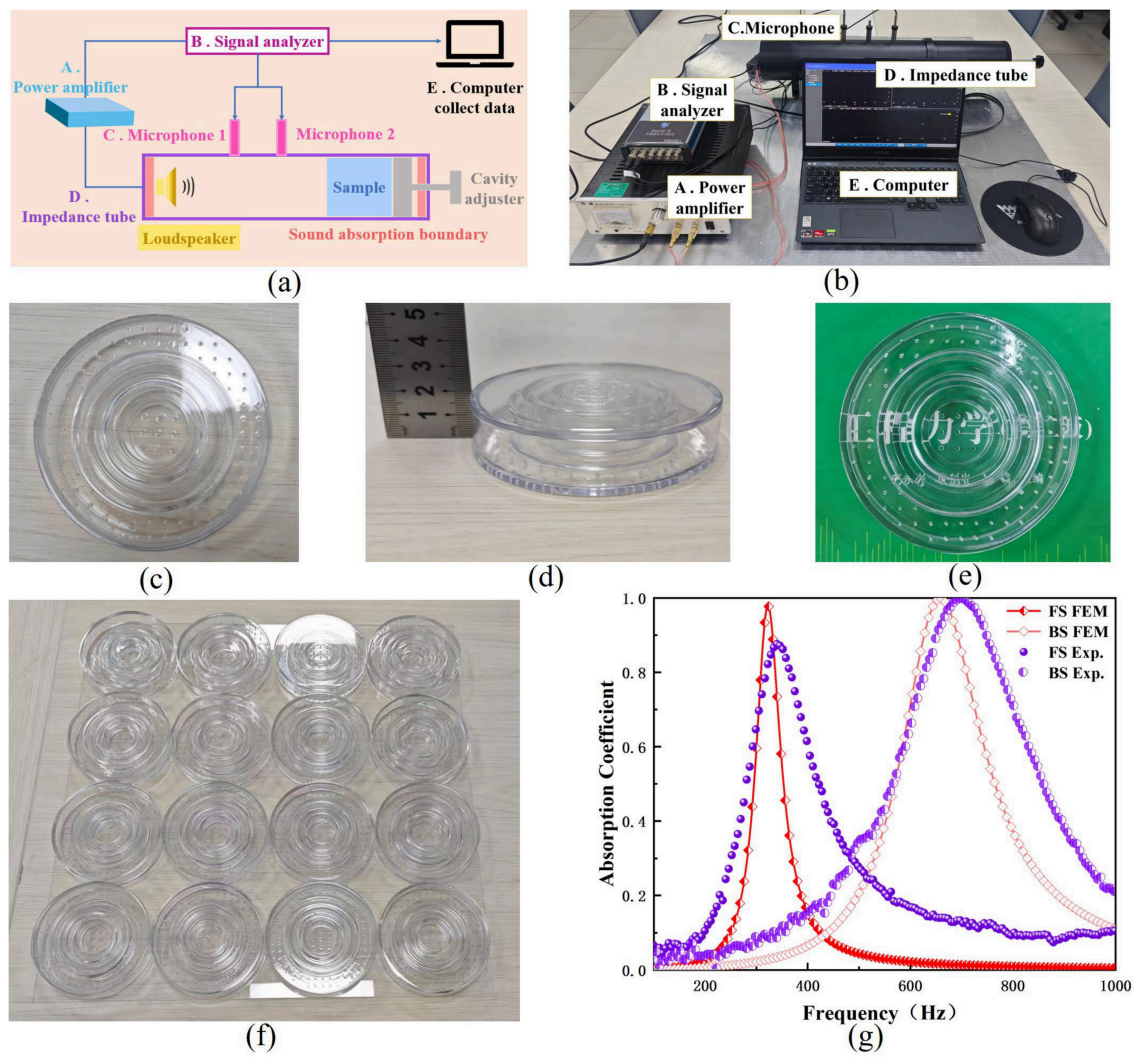
Experimental validation. (**a**) Schematic diagram of impedance tube test procedure; (**b**) photograph of experimental test equipment; (**c**) top view of the fabricated specimen; (**d**) thickness dimension of the test specimen; (**e**) visible-light transparency verification of the transparent specimen. Chinese characters printed on the background book (“Engineering Mechanics”) can be clearly seen through the sample to demonstrate its high transparency; (**f**) broadband sound absorption realization via staggered arrangement of specimen front and back sides; and (**g**) comparison of measured sound absorption results (Exp.) and finite-element simulation results (FEM) of the fabricated specimen.

Prior to the formal test, three sponge pads with a diameter of 10 cm are installed at one end of the impedance tube for equipment calibration. This calibration operation can improve measurement accuracy and reduce systematic experimental errors. After calibration, the printed specimen is placed inside the impedance tube. White noise generated by the data acquisition front-end is transmitted to the loudspeaker through a power amplifier, and the internal sound pressure signals are captured by high-sensitivity microphone sensors. Collected acoustic signals are transmitted to the acoustic analyzer, and experimental sound absorption data are calculated and processed via supporting computer software. The whole experimental procedure is displayed in Figure 8a, and the experimental test platform is presented in Figure 8b.

The sound absorption mechanism of the proposed metastructure is determined by topological structural characteristics rather than material properties, so the prototype can be regarded as a rigid body. The transparent SLA resin adopted in this experiment belongs to a typical rigid material, and the structural vibration effect can be neglected during acoustic testing. The characteristic acoustic impedance of air is 400 kg/(m^2^·s), and the acoustic impedance of SLA resin is 2.53 × 10^6^ kg/(m^2^·s). The acoustic impedance mismatch exists at the medium interface. Larger impedance mismatch leads to stronger acoustic energy reflection at the boundary. Since the acoustic impedance of SLA resin is far higher than that of air, the structural vibration interference can be completely ignored. In addition, all geometric parameters of the printed specimen are consistent with the designed structural parameters. The fabricated experimental specimens are exhibited in Figure 8c–f.

In this study, the impedance tube method is adopted to test the sound absorption performance of the specimen. A rigid sealed wall is arranged at the back of the sample to eliminate sound transmission through the structure. The incident acoustic energy is only distributed between acoustic reflection and internal thermoviscous dissipation. Therefore, the sound absorption coefficient is selected as the core index to evaluate the noise-reduction performance of the resonant acoustic metastructure. The measured sound absorption curves of the specimen are presented in Figure 8g. Although slight mismatches exist between experimental data and numerical results, the overall curve trends maintain high consistency. The resonant frequency and variation tendency of sound absorption curves under the two incident directions match well between simulation and experiment, with only minor deviation in peak amplitude, which verifies the accuracy of the established finite-element model. The distinct difference in sound absorption frequency bands under the two working conditions fully confirms the bidirectional asymmetric acoustic response of the proposed Janus metastructure, which can realize low-frequency narrowband noise reduction and mid-to-high frequency broadband noise suppression separately. The discrepancies between simulation and experimental results are mainly caused by four factors [38,39]: (1) The experimental specimen is assembled by glued components, and excessive adhesive at bonding joints increases the actual structural size and changes the effective inner cavity volume; (2) Poor interfacial bonding occurs during gluing operation, resulting in uneven inner wall surfaces of the metastructure. Besides, the rough surface of 3D-printed SLA resin enhances additional acoustic dissipation, making partial experimental absorption values higher than simulated data; (3) Manufacturing precision errors lead to deviations in actual perforation diameter and hole spacing, which affects the overall acoustic performance; (4) Ineliminable systematic errors exist in the acoustic measurement system, which will not cause obvious variation of sound absorption coefficients.

## 7. Conclusions

In this study, we design and fabricate a composite-perforated Janus acoustic metastructure based on transparent SLA resin. The bidirectional sound absorption mechanism and acoustic regulation performance are systematically investigated via theoretical analysis, finite-element simulation, parametric optimization, and impedance-tube experimental tests. The research results demonstrate that near-perfect low-frequency narrowband sound absorption can be achieved around 322 Hz under frontal incidence from the central perforated panel, while efficient broadband sound absorption is obtained at 658 Hz under backward incidence from the annular micro-perforated panel. Obvious differences in sound absorption characteristics under the two working conditions fully verify the Janus asymmetric acoustic response of the proposed structure. Moreover, the sound absorption curves acquired from theoretical calculation, numerical simulation and experimental measurement achieve great consistency, which confirms the reliability of the adopted research methods. The front perforation parameters mainly modulate the Helmholtz resonant frequency, while the sound absorption performance of the back micro-perforated structure is highly sensitive to aperture size. The sound absorption frequency bands on two sides can be independently tuned by adjusting bilateral structural parameters, presenting excellent acoustic tunability. Compared with conventional single perforated or micro-perforated sound-absorbing structures, the proposed design integrates two types of noise-reduction mechanisms in a single unit (relying on the coupled configuration of multi-stage annular labyrinth channels) which breaks through the limitations of single function and poor low-frequency noise-reduction capacity of traditional structures. Fabricated via SLA resin 3D printing, the designed metastructure integrates visible-light transparency and excellent noise-reduction performance. It possesses broad application prospects in daylighting equipment, industrial pipelines, indoor noise reduction, and other scenarios requiring both light transmission and noise control.

Although the integrally printed transparent acoustic metamaterial achieves bidirectional asymmetric broadband low-frequency sound absorption, several limitations still exist in the current research. First, the sound absorption performance is optimized for normal incident sound waves, and the absorption efficiency will deteriorate evidently under oblique incidence. Second, the effective absorption bandwidth is restricted by the fixed geometric parameters of labyrinthine Helmholtz channels; active tuning capability is not realized in the passive structure. Third, this study mainly focuses on room-temperature laboratory tests, while the acoustic behavior under variable temperature, humidity, and long-term service aging has not been investigated. In addition, large-scale manufacturing of large-area transparent metamaterial panels still faces difficulties via SLA printing.

## Figures and Tables

**Table 1:** Material parameters table.

Materials	Poisson’s Ratio	Density (${g/\mathrm{cm}}^{3}$)	Young’s Modulus (GPa)
SLA Resin	0.41	1.15	2.4

## Data Availability

The original contributions presented in the study are included in the article, further inquiries can be directed to the corresponding author.
